# Reduced Immunosenescence of Peripheral Blood T Cells in Parkinson’s Disease with CMV Infection Background

**DOI:** 10.3390/ijms222313119

**Published:** 2021-12-04

**Authors:** Julia D. Vavilova, Anna A. Boyko, Natalya V. Ponomareva, Vitaly F. Fokin, Ekaterina Y. Fedotova, Maria A. Streltsova, Sofya A. Kust, Maria V. Grechikhina, Ekaterina V. Bril, Olga S. Zimnyakova, Elena I. Kovalenko, Alexander M. Sapozhnikov

**Affiliations:** 1 Shemyakin and Ovchinnikov Institute of Bioorganic Chemistry, Russian Academy of Sciences, 117997 Moscow, Russia; Juliateterina12@gmail.com (J.D.V.); boyko_anna@mail.ru (A.A.B.); mstreltsova@mail.ru (M.A.S.); sonya.erokhina@gmail.com (S.A.K.); marygrec@mail.ru (M.V.G.); amsap@mail.ru (A.M.S.); 2 Research Center of Neurology, 125367 Moscow, Russia; ponomare@yandex.ru (N.V.P.); fvf@mail.ru (V.F.F.); ekfedotova@gmail.com (E.Y.F.); 3 Burnasyan Federal Medical Biophysical Center of Federal Medical Biological Agency, 123098 Moscow, Russia; e.brill@inbox.ru (E.V.B.); oz-83@yandex.ru (O.S.Z.)

**Keywords:** Parkinson’s disease, cytomegalovirus, peripheral immune system, neuroinflammation, differentiation of immune cells

## Abstract

Immunosenescence is a process of remodeling the immune system under the influence of chronic inflammation during aging. Parkinson’s disease (PD) is a common age-associated neurodegenerative disorder and is frequently accompanied by neuroinflammation. On the other hand, cytomegalovirus (CMV), one of the most spread infections in humans, may induce chronic inflammation which contributes to immunosenescence, differentiation and the inflation of T cells and NK cells. Currently, there is no clear understanding of immunosenescence severity in PD patients infected with CMV. In this study, we analyzed differentiation stages and immunosenescence characteristics of T cells and NK cells in 31 patients with mild and moderate PD severity, 33 age-matched and 30 young healthy donors. The PD patients were 100% CMV-seropositive compared to 76% age-matched and 73% young CMV-infected healthy donors. The proportion of effector memory T cells re-expressing CD45RA, CD57^+^CD56^−^ T cells and CD57^+^CD56^+^ T cells was significantly reduced in PD patients compared with CMV-seropositive age-matched healthy individuals. The CD57^+^CD56^−^ T cell proportion in PD patients was similar to that of CMV-seropositive young healthy donors. Thus, PD is characterized by reduced peripheral blood T cell immunosenescence, even against the background of CMV infection.

## 1. Introduction

Parkinson’s disease is the second most common neurodegenerative disorder globally after Alzheimer’s disease, which affects 1% of the global population over 60 years of age [1]. PD is a “protein misfolding” disease—the protein α-synuclein undergoes modification which results in Lewy body formation in dopaminergic neurons, and, finally in the neuron loss in the pars compacta of the substantia nigra, and to a lesser extent, in other regions of the brain [2]. Such localization provokes impairment of the neuron dopaminergic activity in these areas leading to a lack of movement control and coordination. The clinical picture of PD is characterized by motor features such as bradykinesia, rigidity, tremor, postural instability, and a wide range of non-motor symptoms (mental, autonomic, sensory, cognitive, etc.) [3]. Currently, great attention is focused on searching for associations between the neurophysiological PD manifestation and peripheral blood indicators (biochemical, cytological, and immunological), which could be used as a diagnostic and/or prognostic marker for the disease [4,5,6].

It was determined that PD development is associated with the processes of chronic inflammation in the brain tissue—neuroinflammation [7,8]. Whether the neuroinflammation is a provoking factor or a result of the neurodegenerative progression is still unknown [9,10]. The fact is that neuroinflammation is accompanied by chronic systemic inflammation during PD, and the aggravation of neurodegeneration was shown in the presence of peripheral inflammation [11,12,13]. Conversely, the activated microglia in PD itself can be a source of pro-inflammatory mediators contributing to the neurotoxic effects and aggravating neurodegeneration [14,15]. On the other hand, it can be assumed that brain cells can react to the signals from the peripheral immunocompetent cells, for example, upon the systemic activation of the cytokine network [16,17].

The central nervous system (CNS) is traditionally considered immunologically privileged, protected from pathogens and immune cells entry by the blood-brain barrier (BBB). However, the communication between the CNS and periphery appears to be more fluid than previously believed [18]. The increased BBB permeability observed in PD can promote the infiltration of the brain with activated monocytes, macrophages, and T-lymphocytes in the area of neuroinflammation [19]. In a mouse model of PD, the infiltration of the brain with T cells was demonstrated, and increased neurodegeneration in the presence of peripheral inflammation was shown [9]. Additionally, T lymphocytes (CD4^+^ and CD8^+^) were detected in postmortem brain samples in PD patients [13]. There is evidence that the peripheral immune system cells can reflect the processes occurring in the brain of PD. For example, the impairment of dopamine synthesis, α-synuclein metabolism, and some altered mitochondrial functions were found in peripheral T cells during PD [4,20,21]. The development of the PD is accompanied by changes in the ratio of the main subsets of circulating lymphocytes, but the observed published data is still inconsistent [22,23,24].

Some of the infections may be considered as possible trigger mechanisms of neuroinflammation. It is assumed that infectious agents may cause initial sensitization of the brain. Several viruses, including influenza, Coxsackie and herpes, have been associated with acute and chronic parkinsonism [25,26,27]. Cytomegalovirus (CMV) infection has been studied in the context of CNS damage. Notably, an association between the presence of CMV and Alzheimer’s disease has been found [28]. Neurotropic viruses induce CNS inflammation either by entering the brain through a damaged BBB or along the peripheral nerves or by activating the innate and adaptive host immune system in the periphery [29]. Studies on CMV-affected brain tissues imply that the basal ganglia, diencephalon and brainstem are the significant sites of CMV localization [30]. 

The proportion of the human population infected with CMV depends on socioeconomic status, with a level between 60 and 70% in industrialized countries [31]. Effective protection against CMV requires the participation of NK cells and T lymphocytes. In elderly subjects as many as 50% of CD8^+^ and 30% of CD4+ T cells can be CMV-specific at the expense of the naïve and other more highly diversified memory T cells [31]. 

Regarding the effect of CMV infection on the immune system, it has been shown that CMV can stimulate replicative senescence of T cells [32]. T cells replicate multiple times due to repeated stimulation with pathogens during infection, then differentiate, lose their proliferation capacity and may reach the stage of replicative senescence [33]. T cell replicative senescence is a terminal state characterized by dysregulated immune function, loss of the CD28 costimulatory molecule, shortened telomeres, and elevated production of pro-inflammatory cytokines [34]. Additionally, higher levels of effector memory T cells (T_EM_) and effector memory T cells re-expressing CD45RA (T_EMRA_) cells were found in the CD4^+^ and the CD8^+^ T cell pools in CMV-seropositive elderly compared to CMV-seronegative elderly donors [35]. 

CMV infection induces massive inflation of terminally differentiated CMV-specific CD8^+^ T cells and an expansion of adaptive-like NK cells characterized by the expression of the activating receptor NKG2C and the marker CD57 [36]. These changes may lead to clinical consequences including, neurodegeneration in the elderly [31]. It has been shown that NKG2C^+^ NK cells regulate the expansion of CMV-specific CD8+ T cells: depletion of NKG2C^+^ NK cells augments expansion of CMV-specific CD8^+^ T cells [36]. Interestingly, in both CD56^+^ and CD56^−^ subsets, most of the NKG2C^+^ T cells had a phenotype of highly differentiated CD8^+^ T_EMRA_ cells [37]. Furthermore, an association between CMV-specific CD8^+^ T-cell responsiveness and the frequency of NKG2C^+^ NK cells expressing CD57^+^ was detected [38]. 

Aging and many chronic inflammatory diseases induce immunosenescence. To compensate for functional defects of NK and T cells with age, an accumulation of cells combining features of both the innate and adaptive elements of the immune system often occurs [39]. For example, senescent terminally differentiated T_EMRA_ cells acquire features of NK cells, including the upregulation of CD56, a marker used for NK cell identification. These cells in elderly people use the acquired NK cell machinery to maintain rapid effector response against tumor cells and infections [40]. CD56 is also expressed by some γδ T cells. T cells expressing CD56 form a heterogeneous subset of so-called NKT-like (CD3^+^CD56^+^) cells. Alterations in the number, phenotype, and function of immune cells in the CD56^+^ fraction have been reported in patients with various infections [40].

Differentiated T cells often express the CD57 antigen on their surface, a sulfated glycoepitope whose physiological ligands remain unknown. The proliferation capacity of CD57^+^ T cells is severely impaired [33], and CD57 in T lymphocytes is a routinely used marker of replicative senescence [41]. CMV-positivity is associated with changes in the overall T cell repertoire phenotype in healthy aged donors, including an increase in CD57^+^ cell proportion and a decrease in CD28^+^ and CD27^+^ expression, i.e., a phenotypic profile characteristic of immune senescence [42]. Interestingly, in the CD8^+^ T cell fraction from PD patients, a reduced percentage of the CD57^+^ cells and an increased proportion of CD28^+^ cells were observed [24]. 

Since CMV is a widespread infection, it is important to analyze the impact of CMV on the PD-associated changes in immune cell composition and to estimate the immune senescence characteristics under these factors. In this study, we identified differentiation stages of the main subsets represented in the peripheral blood (T cells, NK cells and NKT-like cells) during PD and analyzed changes in this repertoire in relation to CMV infection.

## 2. Results

### 2.1. Analysis of the Alterations in the Ratio of Peripheral T Cells and NK Cells between PD Patients and HD Group in the Context of CMV Persistence

Patients with mild severity and moderate severity PD (31 PD patients) and age-matched healthy donors (33 HD) were recruited in this study (Table 1). 

Assuming the possibility of CMV infection acting as a provoking factor of neurodegenerative progression, we first analyzed the serological CMV status of the PD patients and healthy individuals. The proportions of CMV-infected individuals were 100% (31/31) and 76% (25/33) in the PD patient and HD group, respectively (*p* = 0.003).

Then, we analyzed T and NK cells by flow cytometry in peripheral blood mononuclear cells (PBMC) of recruited individuals independently of their CMV serological status by flow cytometry. There were no significant differences in the T cell (CD3^+^) proportions, including CD4^+^ and CD8^+^ T cell subsets and NK cells (CD3^−^CD56^+^), between PD patients which were all CMV-seropositive and HD groups in our cohorts (Table 2). No differences were found either when we compared the same parameters between PD patients and CMV-positive individuals in HD groups (Table 2). Thus, we revealed no PD-associated changes in the proportions of T cells (CD3^+^), including CD4^+^ and CD8^+^ T cell subsets and NK cells under CMV persistence.

### 2.2. CMV Infection Accompanies the Alterations in the Repertoire of Differentiated Cells in PD Patients

**Figure 1:**
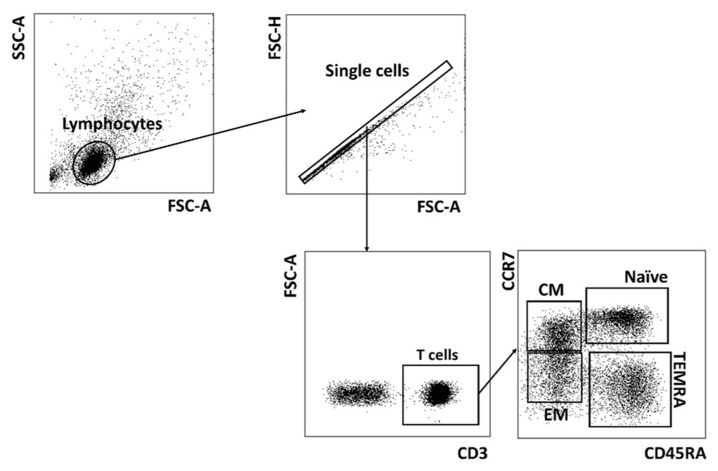
Gating strategy for the T cell naïve/memory subsets. T cells (CD3^+^) at different stages of differentiation: naïve, T_CM_, T_EM_ and T_EMRA_, are selected.

We analyzed T cell repertoire in the context of cell differentiation (naïve and memory cells) in the cohorts of PD patients and HD. We evaluated the percentage of naïve (CCR7^+^CD45RA^+^) T cells, central memory T cells (T_CM_, CCR7^+^CD45RA^−^), effector memory T cells (T_EM_, CCR7^−^CD45RA^−^) and effector memory cells re-expressing CD45RA (T_EMRA_, CCR7^−^CD45RA^+^ T cells) in these groups in relation to CMV infection (Figure 1).

**Figure 2:**
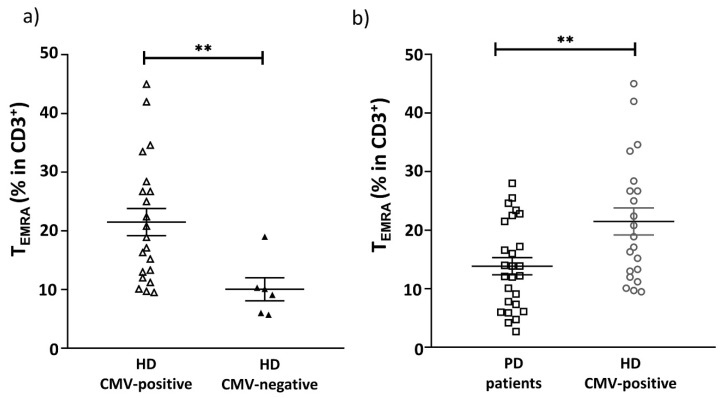
The figure shows the proportion of T_EMRA_ cells among the CD3^+^ cell population. (**a**) The proportion of T_EMRA_ cells in CMV-positive and CMV-negative individuals in the HD group. (**b**) The proportion of T_EMRA_ cells in PD patients and CMV-positive individuals in HD group. Solid line—the mean (±SEM), ** *p* < 0.01.

The percentage of CMV-negative donors in the HD group was 24%, which was in accordance with the average spread of CMV infections in the general population [43]. In our representative HD cohort, we found that the proportion of T_EMRA_ cells in CMV-positive HD individuals was higher than in CMV-negative donors (21.5 vs. 10.03, respectively, *p* = 0.005; taking into account unequal samples), showing the CMV-induced shift of T cell differentiation towards T_EMRA_ cells. There were no significant differences in our cohort’s proportion of naïve and memory T cell subsets (T_CM_, T_EM_, T_EMRA_) between PD patients and HD group (Table 3). At the same time, the proportion of T_EMRA_ cells was significantly reduced in PD patients compared with CMV-positive individuals from HD group (Figure 2b, Table 3). No difference was observed in the proportions of naïve, T_CM_ and T_EM_ cells between PD patients and CMV-positive HD (Table 3).

### 2.3. Replicative Senescence of CD56^−^ and CD56^+^ T Cells Evaluated by CD57 and NKG2C Expression in PD Patients and CMV-Positive HD Cohorts

**Figure 3:**
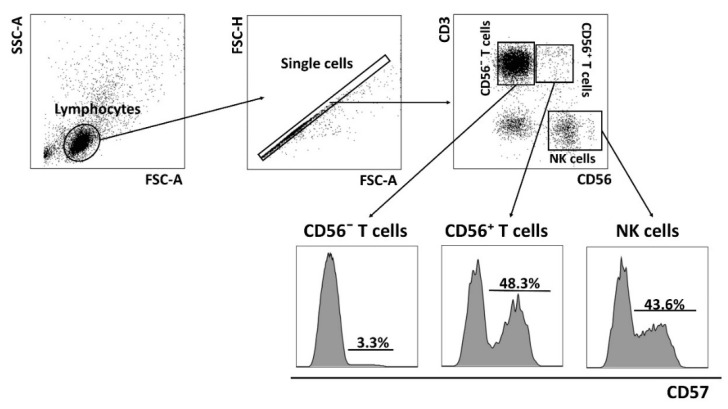
CD57 expression analysis on T cells and NK cells. Gating strategy and representative data of CD57+ cell proportions in CD56^−^ T cells (CD3^+^CD56^−^), CD56^−^ T cells (CD3^+^CD56^+^) and NK cells (CD3^−^CD56^+^) are presented.

One of the features of the senescent T_EMRA_ cells is CD56 expression in part of these cells—the so-called NKT-like cells with CD3^+^CD56^+^ phenotype [40]. Alterations in the level, phenotype and function of immune cells in the CD56^+^ fraction have been reported in patients with various infections [40]. We compared the distribution of CD56^−^ T cell and CD56^+^ T cells and replicative senescence degree in PD patients and the CMV-positive HD group (Figure 3). We revealed no significant differences in the proportion of CD56^+^ and CD56^−^ T cells between these groups in our study (Table 4)

**Figure 4:**
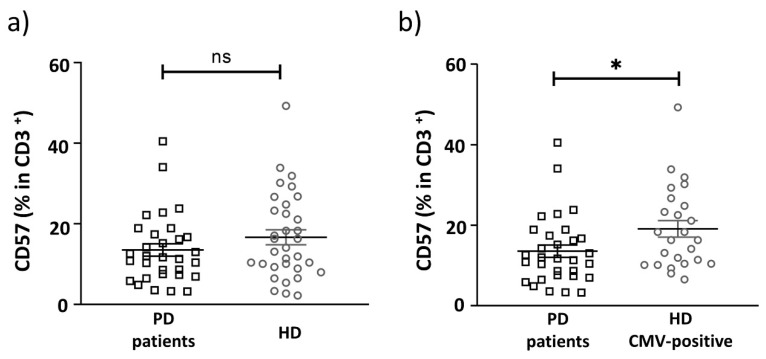
The proportion of CD57^+^ cells among all CD3^+^ T cells in PD patients and HD group. Comparative analysis of the CD57^+^ cell percentage in CD3^+^ T cells: (**a**) between PD patients and HD group, which included CMV-positive and CMV-negative donors; (**b**) between PD patients and only CMV-positive individuals in the HD group. Solid line—the mean (± SEM). n.s. non-significant, * *p* < 0.05.

Upon CMV infection, the proportion of T cells expressing the CD57 antigen, a marker for identification of terminally differentiated ‘senescent’ lymphocytes [33], is often increased. We measured CD57^+^ T cells in PD patients and the age-matched HD group. The proportion of the CD57^+^ cells among all CD3^+^ T cells was decreased in PD patients compared to CMV-positive individuals from the HD group (Figure 3 and Figure 4, Table 4).

**Figure 5:**
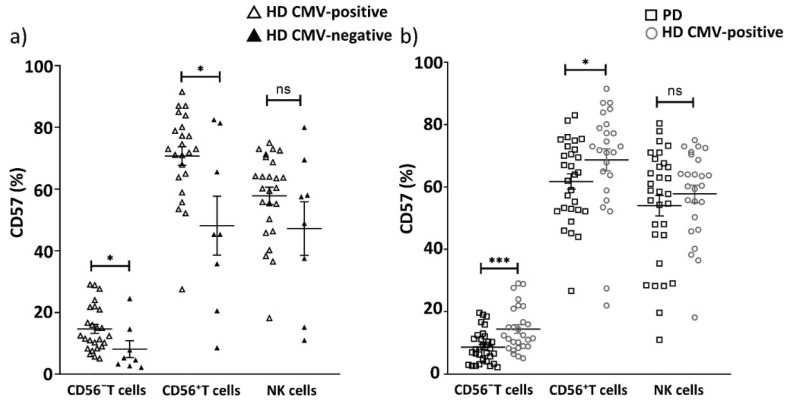
CD57^+^ cells (%) content among T cells and NK cells, depending on CMV infection. Comparing CD57^+^ cell percentage in CD56^−^ T cells, CD56^+^ T cells and NK cells (**a**) between CMV-positive and CMV-negative donors in HD group; (**b**) between PD patients and CMV-positive individuals in HD group. Solid line—the mean (± SEM). n.s. non-significant, * *p* < 0.05, *** *p* < 0.001.

Next, we analyzed more precisely the T cell subsets in which a CD57^+^ cell decrease was observed. We compared CD57^+^ cell percentage in CD56^−^ and CD56^+^ T cell subsets between CMV-positive and CMV-negative donors in the HD group. The proportions of CD57^+^CD56^−^ T cells and CD57^+^CD56^+^ T cells in CMV-positive HDs were higher than in CMV-negative HDs (14.7 vs. 8.1, *p* = 0.01; 70.7 vs. 48.1, *p* = 0.04, respectively) (Figure 5a). At the same time, the proportions of both CD57^+^CD56^−^ T cells and CD57^+^CD56^+^ T cells were lower in the group of PD patients compared with the same marker in the CMV-positive HD group (Figure 5b, Table 4). 

Since CMV infection is associated with an accumulation of replicatively senescent CD57^+^ adaptive-like NK cells [44], we estimated the proportion of CD57^+^ NK cells in our cohorts. No abnormalities in CD57-expressing NK cell proportion were observed in PD patients (Figure 5b, Table 4). 

**Figure 6:**
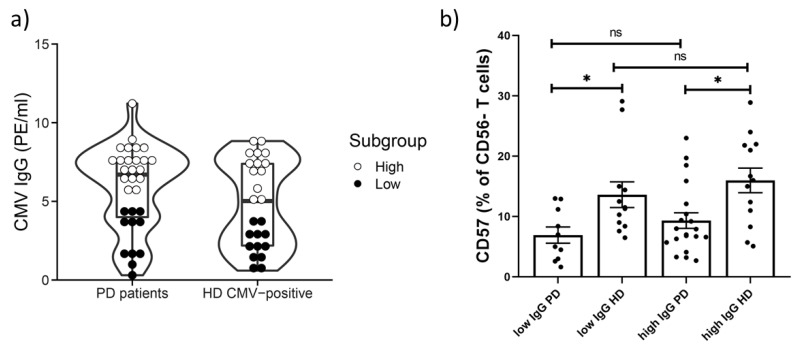
The characteristics of subgroups based on high and low IgG CMV value among PD patients and HDs (**a**) The frequency distribution of CMV IgG value in PD patients and HDs. (**b**) The percentages of CD57^+^ cells among CD56^−^ T cells in PD patients and HD with low and high CMV-specific IgG levels. Solid line—the mean (±SEM). n.s. non-significant, * *p* < 0.05.

Then, PD patients and CMV-positive individuals from HD groups were divided into subgroups with low and high IgG values. The following subgroups were formed: low IgG PD; high IgG PD; low IgG HD; high IgG HD (Figure 6a). In both low and high IgG PD patient subgroups a reduced proportion of CD57^+^ cells in the CD56^−^ T cell subset compared to low and high IgG HD subgroups was observed (6.9 vs. 13.6, *p* = 0.02; 9.3 vs. 15.9, *p* = 0.01, respectively). However, no differences in the proportion of CD57^+^ cells in CD56^−^ T cells between low and high IgG subgroups were found between the PD patient group and in the HD group (6.9 vs. 9.3, *p* = 0.3) (13.6 vs. 15.9, *p* = 0.4) (Figure 6b). Thus, we confirmed that the CD57^+^CD56^−^ T cell subset decreased in PD patients compared with CMV-matched HDs. This reduction was independent of CMV IgG values.

**Figure 7:**
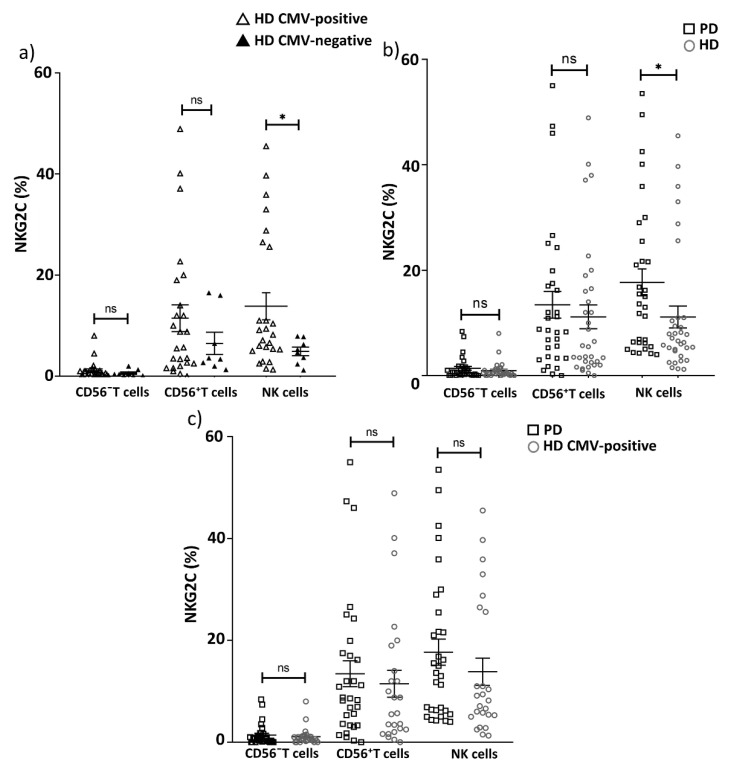
The proportion of NKG2C^+^ (%) cells among CD56^−^ T cells, CD56^+^ T cells and NK cells, in relation to CMV infection. Differences in the level of NKG2C^+^ cells (%) in CD56^−^ T cells, CD56^+^ T cells and NK cells between (**a**) the groups of CMV-positive and CMV-negative HDs; (**b**) between PD patients and HD group (CMV-positive and CMV-negative individuals combined); (**c**) between PD patients and only CMV-positive HDs. Solid line—the mean (±SEM). n.s. non-significant, * *p* < 0.05.

Since the immune response to CMV often leads to the accumulation of T and adaptive-like NK cells expressing the activating receptor NKG2C [44], we analyzed the proportion of the NKG2C^+^ cells in the CD56^−^ and CD56^+^ T cell fractions and in the NK cells in PD patients and the HD group. As expected, the proportion of NKG2C^+^ NK cells was higher in CMV-positive donors then in CMV-negative donors in the HD group (13.9 vs. 4.9, respectively, *p* = 0.04). There was a tendency towards an increased level of NKG2C-expressing T cells, especially, the CD56^+^ T cells subset in CMV-positive HD compared to CMV-negative HD (Figure 7a), although the difference was not significant, possibly, due to the small size of CMV-negative group. Next, we compared the proportion of cells expressing the activating receptor NKG2C in the CD56^−^ T cells, CD56^+^ T cells and NK cells subsets in PD patients and the HD group. The proportion of NKG2C^+^ NK cells was higher in PD patients than in the HD group (Figure 7b, Table 4), which was not surprising because individuals with PD were 100% infected with CMV. At the same time, no difference in NKG2C^+^ NK cell level was found between healthy CMV-positive individuals and the PD patient group. No alterations in the proportion of NKG2C^+^ cells among CD56^−^ and CD56^+^ T cell subsets between CMV-positive cohorts of HDs and PD patients were found (Figure 7c). Thus, the CMV-induced formation of NKG2C-expressing subsets of NK cells and T lymphocytes was not altered in PD.

### 2.4. Age-Related Increase in CD57^+^CD56^−^ T Cells Subset Not Detected in PD Patients

To evaluate the influence of age on the amount of CD57^+^CD56^−^ T cells, we additionally enrolled 30 young healthy donors (YHD) (median aged—25 ± 2.3) into our investigation. In total, 73% (22/30) of the YHD donors were CMV-positive, which differed significantly from the PD group in terms of CMV-seropositivity (*p* = 0.001). 

**Figure 8:**
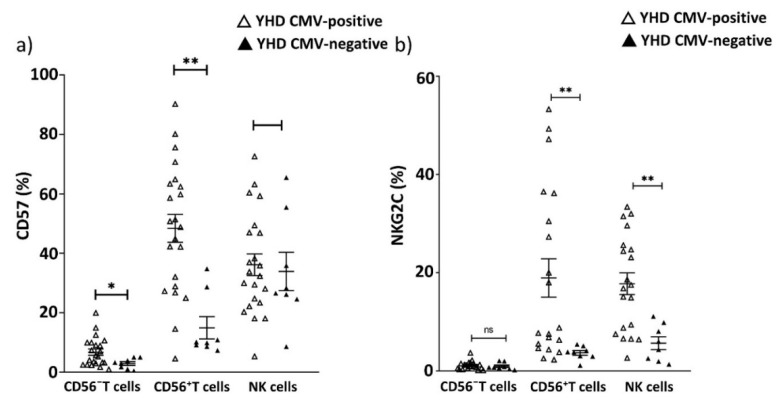
The proportion of CD57^+^ and NKG2C^+^ cells in CMV-positive and CMV-negative young healthy individuals: (**a**) The level (%) of CD57^+^ cells among CD56^−^ and CD56^+^ T cells and NK cells; (**b**) Percentages of NKG2C^+^ cells in CD56^−^ T cells, in CD56^+^ T cells and in NK cell subsets. Solid line—the mean (±SEM). n.s. non-significant, * *p* < 0.05, ** *p* < 0.01.

The proportions of CD57^+^CD56^−^ T cells and CD57^+^CD56^+^ T cells in CMV-positive YHD donors were higher than in CMV-negative YHD donors (6.7 vs. 2.3, *p* = 0.01; 48.4 vs. 14.9, *p* = 0.0003, respectively), similarly to the HD group described above (Figure 8a). In addition, the increased proportion of NKG2C+ cells among CD56^+^ T cells and in NK cells in the CMV-positive YHD group vs. CMV-negative YHD group was shown (18.9 vs. 3.6, *p* = 0.002; 17.8 vs. 5.6, *p* = 0.003, respectively) (Figure 8b).

**Figure 9:**
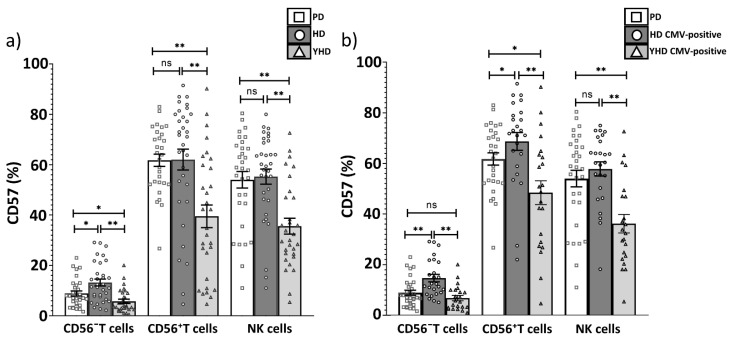
The content of CD57^+^ (%) in CD56^−^ T cells, CD56^+^ T cells and NK cells in PD patients, HD and YHD concerning CMV persistence: (**a**) between the three comparison groups including CMV-positive and CMV-negative individuals; (**b**) between only CMV-positive individuals from PD patients, HD and YHD groups. Solid line—the mean (±SEM). n.s. non-significant, * *p* < 0.05,** *p* < 0.01.

As expected, the proportion of CD57^+^ cells was lower in both CD56^−^ and CD56^+^ T cell subsets, and in NK cells in the group of YHD compared with PD patients and HDs groups (Figure 9a). As we described above, the proportions of CD57^+^ cells in CD56^−^ T cell was reduced in PD patients vs. CMV-positive healthy individuals. Interestingly, there were no significant differences in the proportion of CD57^+^CD56^−^ T cells in the PD patients vs. CMV-positive individuals from the YHD group (7.0 vs. 5.5, *p* = 0.2). At the same time the proportions of CD57^+^CD56^+^ T cells and CD57^+^ NK cells has remained increased in the PD patients vs. CMV-positive individuals from YHDs (61.8 vs. 48.4, *p* = 0.02; 54.0 vs. 36.2, *p* = 0.0007, respectively). (Figure 9b). Thus, the phenomenon of the reduction in CD57^+^CD56^−^ T cells in PD patients was not mediated by age-related components.

## 3. Discussion

The involvement of several viruses in PD pathogenesis has been described [29,45]. However, there is little data on the role of cytomegalovirus in PD. CMV is one of the most widespread viruses in the human population. Proportions of CMV-seropositive individuals in human populations vary in different countries ranging approximately from 50% to 100% [43]. In our study, in the control group of HD, the proportion of CMV-positive individuals was 76%, which is typical for the Russian population. However, all individuals in the PD patients group were CMV-seropositive. This might imply an association between CMV infection and PD development. At the same time, the data on the increased proportion of CMV-infected persons within PD patients need to be confirmed in larger cohorts. 

T cells infiltrate the CNS during neuroinflammation, which has been described previously [13]. Lymphocytes are considered to limit neuronal damage caused by infection, mechanical injury, or neurodegenerative processes [46]. PD patients were shown to have increased permeability of BBB, which promotes cytokines, chemokines, and damage-associated soluble mediators of systemic inflammation, gaining access to the CNS from the blood flow through the damaged the BBB [47,48]. However, the communication between circulating blood T cells and the CNS in PD is still poorly described. Potentially, CMV can contribute to lymphocyte brain infiltration and provoke PD by affecting the differentiation of both T and NK cells, driving functional and phenotypic changes in the repertoires of these cells and inducing chronic inflammation [34,35,49].

In this study on PD, we focused on differentiation and senescence characteristics of peripheral lymphocytes, which play a significant role in antiviral defense, in PD and on their relevance of CMV persistence.

It is known that CMV seropositivity is associated with changes in the phenotype of the overall T cell repertoire in healthy aged donors [50]. Initially, we analyzed T cells at distinct differentiation stages according to the standard scheme, which includes naive T cells and cells after activation – effector T cells and memory T cells, capable of rapid reactivation. Memory T cells represent a heterogeneous cell population that are divided into several subsets using CD45RA and CCR7 expressed on the cell surface markers. CD45RA^−^CCR7^+^ central memory (TCM) cells possess a high self-renewal potential, but low secretory activity, while T cells with the CCR7^−^ phenotype form two subsets: effector memory T cells (TEM, CCR7^−^CD45RA^−^) and T effector memory cells re-expressing CD45RA (T_EMRA_) cells (CCR7^−^CD45RA^+^), characterized by a limited proliferative capacity but higher secretion levels of a wider range of cytokines after activation [51]. In our study, no significant differences were revealed between the level of naïve, T_CM_, T_EM_ and T_EMRA_ subsets of T cells (CD3+) in peripheral blood of PD patients and the HD group. It is known that CMV seropositivity determines the increase in CD8^+^ T_EMRA_ cells [31], which was confirmed in our HD cohort. There was a shift in T cell differentiation towards T_EMRA_ cells in CMV-positive individuals (Figure 2a). Interestingly, among only the CMV-positive individuals, the CD3^+^ T_EMRA_ cells subset in PD patients was significantly reduced compared with the HD group (Figure 2b, Table 3). 

CD56 expression is usually increased at the late stages of T cell differentiation (in so-called NKT-like cells). Various infections can provoke the expansion of CD56^+^ T subsets [40]. So, we assessed the distribution of CD56^−^ T cells and a more differentiated CD56^+^ T cell subset in PD. We did not find any proportional differences in CD56^−^ T cell and CD56^+^ T subsets between PD patients and the HD group nor among only CMV-positive individuals from these groups. This fact indicates that more differentiated CD56^+^ T cells (NKT-like cells), which share some phenotypical and functional features with NK cells [37], apparently do not participate in PD-mediated response. 

In our healthy cohorts of young and elderly donors (YHD and HD), we registered an age-associated increase in the proportion of senescent CD57^+^ cells in accordance with the data reported previously [52]. CMV-positive individuals demonstrated higher levels of CD57^+^ cells in CD56^−^ and CD56^+^ T cell subsets than CMV-negative donors from YHD and HD groups (Figure 5a and Figure 8a). On the other hand, in PD patients, the proportions of CD57^+^ T cells were decreased compared to age-matched HDs against the background of CMV infection (Figure 9b). The difference in the size of CD57^+^ T cell subset did not depend on serum CMV-specific IgG level, thus, it did not depend on time and/or intensity of CMV-reactivation. At the same time, it does not exclude a potential role of CMV infection at initial stages of PD. Abnormalities of age-related T cells senescence in PD was shown earlier [24]. We have shown that the level of CD57 expressing T cells was reduced more profoundly in conventional CD56^–^ T cells from PD patients, and was similar to the level in CMV-seropositive YHDs (Figure 9b). Despite the CMV infection-causing reconfiguration of the NK cells repertoire, including accumulation of CMV-associated adaptive-like NK cells expressing activating receptor NKG2C [53,54], no reduction in CD57^+^ NK cells in PD patients was observed in our study. We did not find any differences in the NKG2C surface levels in the NK cell population nor in T cells, in which elevated NKG2C expression is often associated with CMV-induced the T cell inflation [36]. So, the NK and T cell NKG2C^−^ associated responses apparently are not altered in PD.

There are several possible explanations for the decrease in CD57^+^ T cell content in PD patients. First, CD57^+^CD56^−^ and CD57^+^CD56^+^ T cells in PD upon CMV might more intensively migrate from the bloodstream into different tissues Enhanced migratory potencies of senescent CD8^+^CD57^+^ T cells to nonlymphoid tissues mediated by chemokine receptor CX3CR1 have been shown earlier [55]. It was suggested that CD8^+^CD57^+^ effector cells expressing CX3CR1 are recruited into the brain of HIV-infected patients under the up-regulation of CX3CL1 in astrocytes from patients with acquired immunodeficiency syndrome dementia [56]. Neuroinflammation and BBB disruption can promote T cells infiltration to the affected brain regions during PD [57]. Thus, the increased BBB permeability observed during PD can promote the infiltration of the brain with activated CD57^+^ T cells.

On the other hand, CMV infection is characterized by the accumulation of CMV-specific CD8^+^ T cells with a differentiation pathway distinct from the formation of memory CD8^+^ T cells after infection with acute viruses [58]. Like in T cells, CD57 is expressed in NK cells as a considered marker of terminal differentiation [59]. It was demonstrated in NK cells, that in specific conditions in vitro, terminally differentiated CD57^+^ human NK cells can acquire the CD57^−^ phenotype [60]. So, we can assume that T cells might be also capable of losing the CD57 molecule. It was shown, that the presence of α-synuclein-reactive T cells in PD patients [61] and serum α-synuclein levels were higher during the PD [62]. Thus, we can hypothesize that due to stimulation by a specific antigen, such as α-synuclein, CD57^+^ T cells from PD patients can lose this molecule and become CD57^−^ T cells. The phenomenon of low accumulation of CD57^+^ T cells in response to CMV infection in PD should be considered in further phenotypic evaluation of peripheral lymphocytes of PD patients. 

Presumably, the development of PD might be promoted by the cellular immune response formed during previous or concurrent viral infections. We found that PD is characterized by reduced peripheral T cell immunosenescence, even against the background of CMV infection. These changes in the peripheral immune cells in PD patients may help to determine the focus of further studies on the CNS immune system abnormalities related to neurodegenerative disorders.

## 4. Materials and Methods

### 4.1. Participants and Ethics Statement

This study was approved by the RCN Local Medical Ethics Committee (protocol No. 11/14 from 19 November 2014), and all participants provided written informed consent. The demographic and clinical characteristics of donors and CMV status are shown in Table 1. In total, 31 patients with Parkinson’s disease (PD patients), 33 age-matched healthy donors (HD) and 30 young healthy donors (YHD) were recruited for the study. There were no significant differences in age and gender composition between the groups of HD and PD patients. PD was diagnosed according to the following criteria: “Parkinson’s Disease Society Brain Bank” [63]. The examined donors had idiopathic PD. The stages of the disease were determined on a scale of Hoehn and Yahr. Hoehn and Yahr stage ≤ 3. Clinical symptoms, including movement disorders, were assessed using a unified PD scale—MDS-UPDRS (the Movement Disorder Society—Unified Parkinson’s Disease Rating Scale). All patients were treated with carbidopa/levodopa in combination with dopamine agonist therapy. None received immunosuppressive therapy. PD patients underwent full clinical assessment including medical history and comorbidities. None of the patients had acute infectious or autoimmune disease, identified within a month before the examination. Healthy donors underwent neurological examination and cognitive screening and were found to be free of dementia and other medical, psychiatric, and neurological conditions, including cerebrovascular diseases, hypertension, epilepsy, and endogenous disorders. Exclusion criteria were a history of neurological and psychiatric diseases and any kind of memory impairment.

### 4.2. Isolation of PBMC from Peripheral Donor Blood and the Sample Preparation

Peripheral blood mononuclear cells (PBMC) were isolated using gradient centrifugation with standard ficoll solution (PanEco, Moscow, Russia) (density 1.077). PBMC were washed twice (400× *g*, 15 min) in Dulbecco’s phosphate-buffer saline (DPBS). For surface fluorescent immunostaining cells were incubated with antibodies for 30 min on ice in PBA staining buffer (PBS containing 0.5% BSA (bovine serum albumin) (Serva, Heidelberg, Germany) and 0.01% sodium azide (AMRESCO, Inc. (VWR International, LLC), Aurora, CO, USA) and then washed twice in the same buffer.

### 4.3. Antibodies for Flow Cytometry

The following mouse anti-human fluorescent-labeled antibodies were used for surface cell staining: CD56-APC (clone N901, Beckman Coulter, Miami, FL, USA), CD4-FITC (clone RPA-T4, Sony Biotechnology, San Jose, CA, USA), CD57-PE (clone HCD57, Sony Biotechnology, San Jose, CA, USA), NKG2C-PE (clone 134591, R&D Systems, Minneapolis, MN, USA), CD3-PerCP (clone HIT3a, Sony Biotechnology, San Jose, CA, USA), CD3-FITC (clone FIT3a, Sony Biotechnology, San Jose, CA, USA), CD3-APC (clone F OKT3, Sony Biotechnology, San Jose, CA, USA) CD8-PerCP (clone SK1, Sony Biotechnology, San Jose, CA, USA), CD45RA-APC (clone HI100, Sony Biotechnology, San Jose, CA, USA), CD197-FITC (clone G043H7, Sony Biotechnology, San Jose, CA, USA), CD56-APC-Vio770 (clone REA196, Miltenyi Biotec, Bergisch Gladbach, Germany).

The following panels of fluorochrome-conjugated monoclonal antibodies to immune cell surface markers for the PD patients and HD group were used: CD4-FITC, CD3-PE, CD8-PERCP (panel 1); CD3-FITC, CD57-PE, CD56-APC (panel 2); CD3-FITC, NKG2C-PE, CD56-APC (panel 3); CD197-FITC, CD3-PerCP, CD45RA-APC (panel 4).

The following panel of monoclonal antibodies for the YHD group was used: CD4-FITC, CD57-PE, CD8-PERCP, CD3-APC, CD56-APC-Vio770.

### 4.4. Flow Cytometry and Data Analysis

Flow cytometry analysis of the PBMC samples was carried out on a FACSCalibur flow cytometer (BD Biosciences, Franklin Lakes, NJ, USA) equipped with 488 and 640 nm lasers and an appropriate set of detectors and filters and on a MACSQuant 10 cytometer (Milteniy Biotech, Bergisch Gladbach, Germany) equipped with 405 nm, 488 nm, and 635 nm lasers.

The data obtained were processed and presented using FlowJo X 10.0.7r2 (FlowJo LLC, Ashland, OR, USA). The cytofluorimetric analysis was performed after cell staining with the appropriate monoclonal antibodies. Lymphocytes were discriminated by gating the cells using a forward and side scatter cytogram. Then, single cells were selected using forward-scatter area vs. height. CD56^−^ T cells, CD56^+^ T cells and NK cells subsets were identified by gating on CD56 and CD3. In CD3^+^ cells the following subsets were determined: naïve T cells (CCR7^+^CD45RA^+^), central memory T cells (CCR7^+^CD45RA^−^), effector memory T cells (CCR7^−^CD45RA^−^) and terminal effector memory T cells (CCR7^−^CD45RA^+^).

### 4.5. CMV Serology Status

Venous blood was collected in vacuum tubes containing gel with clot activator and gel (APEXLAB, Moscow, Russian). Tubes were left to clot for 30 min, then centrifugated at 3000 rpm and removed of serum, then stored at −80 °C before analysis for CMV IgG using ELISA kit (Vector-Best, Novosibirsk, Russian) according to the manufacturer’s protocol.

### 4.6. Statistical Analysis

Statistical analysis was performed using GraphPad Prizm (ver. 8.00). Data are presented as mean ± standard error of mean (SEM), * *p* < 0.05, ** *p* < 0.01, *** *p* < 0.001 was considered significant. For data with normal distribution, *t*-tests were performed. For non-normally distributed data a, Mann-Whitney U test was used. To divide data into subgroups, the k-means clustering method using the base R language function ‘kmeans’ with standard parameters was performed.

## Figures and Tables

**Table 1:** The demographic and clinical characteristics of PD patients and HD group.

	PD Patients *n* = 31	HD *n* = 33
Age (Median)	59 ± 11.6	56 ± 11
Male (*n*)	13	12
MDS-UPDRS motor score (mean)	25 ± 8.7	
Hoehn and Yahr (mean)	2.7 ± 0.6	
Duration of PD (mean years)	7 ± 0.8	
CMV IgG (% positive)	100 (31/31)	76 (25/33)

Designation *n* is the number of donors. MDS-UPDRS—Movement Disorder Society-Unified Parkinson’s Disease Rating Scale. ±—standard deviation. Hoehn and Yahr—a scale determining stages of PD.

**Table 2:** The proportion of T cells (CD3^+^), including CD4^+^ and CD8^+^ subsets, and NK cells in PD patients, HD group and CMV-seropositive HD individuals.

Subset (% of Lymphocytes)	PD Patients *n* = 31 ①	HD *n* = 33 ②	CMV-Positive HD *n* = 25 ③	*p*-Value	
				① vs. ②	① vs. ③
CD3^+^	70.7 ± 2.1	70.6 ± 1.8	71.5 ± 2.1	0.9	0.7
CD3^−^CD56^+^	13.9 ± 1.7	12.1 ± 1.3	11.8 ± 1.5	0.5	0.4
CD3^+^CD4^+^CD8^−^	66.6 ± 1.9	63.5 ± 1.9	63.6 ± 2.4	0.2	0.4
CD3^+^CD4^−^CD8^+^	25.5 ± 1.5	27.9 ± 1.8	28.76 ± 2.3	0.3	0.3

Designation *n* is the number of donors. The values shown are mean ± SEM. **①**, **②**, **③**—groups of donors.

**Table 3:** The proportion of naïve and memory T cells (T_CM_, T_EM_ and T_EMRA_) in PD patients compared to HDs and CMV-positive individuals in the HD group.

Subset (% of T Cells)	PD Patients *n* = 27 ①	HD *n* = 27 ②	CMV-Positive HD *n* = 21 ③	*p*-Value	
				① vs. ②	① vs. ③
CCR7^+^CD45RA^+^ (Naïve)	40.1 ± 2.8	37.3 ± 2.7	34.5 ± 3.1	0.5	0.1
CCR7^+^CD45RA^−^ (T_CM_)	29.8 ± 1.9	28.3 ± 1.9	28.4 ± 1.9	0.6	0.6
CCR7^−^CD45RA^−^ (T_EM_)	15.0 ± 1.7	15.3 ± 1.1	15.4 ± 1.3	0.5	0.8
CCR7^−^CD45RA^+^ (T_EMRA_)	13.85 ± 1.8	18.95 ± 2.	21.50 ± 2.3	0.06	0.005

Designation *n* is the number of donors. The values shown are mean ± SEM. **①**, **②**, **③**—groups of donors.

**Table 4:** The proportion of T cells (CD56^−^ and CD56^+^), the level of CD57^+^ and NKG2C^+^ cells among the CD56^−^ T cells, CD56^+^ T cells and NK cells between PD patients, HD group and only CMV-positive individuals from HD group.

Subsets	PD Patients *n* = 31 ①	HD *n* = 33 ②	CMV-Positive HD *n* = 25 ③	*p*-Value	
				① vs. ②	① vs. ③
CD56^−^ T cells	52.4 ± 2.2	52.9 ± 2.4	53.63 ± 2.7	0.9	0.6
CD56^+^ T cells	3.9 ± 0.5	4.1 ± 0.6	4.7 ± 0.7	0.9	0.6
CD57 (% of CD3^+^ cells)	13.52 ± 1.5	16.7 ± 1.9	19.1 ± 2.1	0.2	0.03
CD57 (% of CD56^+^ T cells)	61.8 ± 2.4	62.1 ± 4.1	68.7 ± 3.5	0.3	0.03
CD57 (% of NK)	54.0 ± 3.2	55.2 ± 3.0	57.8 ± 2.8	0.8	0.5
NKG2C (% of CD56^−^ T cells)	1.3 ± 0.4	0.9 ± 0.3	1.1 ± 0.3	0.3	0.6
NKG2C (%of CD56^+^ T cells)	13.5 ± 2.5	11.6 ± 2.3	11.5 ± 2.5	0.5	0.6
NKG2C (% of NK cells)	17.7 ± 2.6	11.1 ± 2.1	13.8 ± 2.7	0.05	0.3

①-PD patients, ②-HD group, ③-CMV-positive HD group.
